# The role of spiritual care management – Needs and resources in people with amyotrophic lateral sclerosis: Insights from a mixed-methods study

**DOI:** 10.1017/S1478951525000495

**Published:** 2025-05-30

**Authors:** Filipe Gonçalves, Margarida I. Teixeira, Francisca Rego, Bruno Magalhães

**Affiliations:** 1 University of A Coruña, Faculty of Health Sciences, Campuz de Oza, A Coruña, Spain; 2 Department of Rehabilitation and Palliative Care, APELA – Portuguese Association of Amyotrophic Lateral Sclerosis, Porto, Portugal; 3 Oncology Nursing Research Unit IPO Porto Research Center (CI-IPOP), and Portuguese Oncology Institute of Porto (IPO Porto)/Porto Comprehensive Cancer Centre (Porto.CCC) & RISE@CI-IPOP (Health Research Network) & RISE@CI-IPOP (Health Research Network), Porto, Portugal; 4 Faculty of Medicine, University of Porto (FMUP), Porto, Portugal; 5 School of Health, University of Trás-os-Montes and Alto Douro (ESS-UTAD), Vila Real, Portugal; 6 RISE@UTAD-Health Research Network, Faculty of Medicine, University of Porto, Porto, Portugal; 7 Clinical Academic Centre of Trás-os-Montes and Alto Douro (CACTMAD), Vila Real, Portugal

**Keywords:** Amyotrophic lateral sclerosis, motor neuron diseases, spirituality needs and resources, life purpose, palliative care

## Abstract

**Objectives:**

To explore the spiritual needs and resources of People with Amyotrophic Lateral Sclerosis (PALS) at different stages of its trajectory and to characterize the experiences of the current state of the disease.

**Methods:**

A convergent mixed-methods study was conducted using qualitative and quantitative approaches. Participants were assessed using the clinical and sociodemographic data, ALSFRS-R (function assessment), and the GES Questionnaire to evaluate spiritual needs and resources. Data were collected through in-person or online interviews, transcribed and coded. The qualitative analysis was based on the content analysis method. Statistical analysis was performed using SPSS software. Both datasets were integrated during data analysis.

**Results:**

Twenty-four patients were interviewed, with a duration of the illness ranging from 1 year to 12 years. Participants were at different stages of functional dependence. Analyzing the open questions of the GES questionnaire, six categories were established related to the inner world of PALS: *Concern, Nuisance, Help, Support, Safety*, and *Valorization*. Contrary to what was hypothesized, no correlations were found between functionality and the spiritual dimensions. Spiritual needs and resources tend to vary with age, with younger ages presenting a more fragile spiritual dimension overall. Also, the intrapersonal and interpersonal dimension seems to play a central role in the lives of PALS. A negative correlation was identified between the feeling of connection to a supreme/transcendent reality and the level of educational qualifications.

**Significance of results:**

Spirituality often provides crucial emotional support, meaning, and resilience during challenging times. Despite its importance, it is often overlooked in clinical settings. The study emphasizes the need for personalized, holistic care, which should include spiritual care support, regardless of the functional state, highlighting the importance of addressing both intrapersonal and interpersonal domains, resources and needs from early phases. Allowing to create a structured care plan that meets patients’ individual spiritual needs, that can contribute to a better QoL and reduce suffering.

## Introduction

Amyotrophic lateral sclerosis (ALS) is an incurable neurodegenerative disease characterized by motor neuron degeneration (Hardiman et al. 2017). Patients often perceive the disease as an interruption in their lives due to the functional losses that occur during the disease. As a result, ALS presents numerous and profound challenges in all areas of life. In addition to the initial impact of the diagnosis and its prognosis, there are constant adaptations, concerns, and feelings of uncertainty about the future, particularly about the final stages of the disease (Brown and Addington-Hall 2008; Kiernan et al. 2011).

During these times of distress, spirituality (a complex multidimensional concept) plays an important role, offering emotional comfort, meaning, and resilience (Bryson 2004; Moberg 2002). Spirituality becomes a vital resource for coping with difficulties when searching for purpose, relating to beliefs, accepting illness situations, and preparing for death (Gijsberts et al. 2019), highlighting its three dimensions: the intrapersonal dimension reflects the need formeaning and coherence in one’s relationship with himself or herself; interpersonal dimensions include harmony in relationships with significant people and the need to feel loved and loved; and the transpersonal dimension includes hope, the need to leave a legacy beyond oneself, and a relationship with the transcendent (Benito et al. 2014; Manso et al. 2020).

Understanding spiritual care is key in palliative care, as it represents a process involving several organically linked phases that include the identification and knowledge of the patient’s specific spiritual needs and a deep exploration of existing resources that can lead to the development of the individual spiritual care treatment plan, its provision, and reassessment by trained healthcare and/or spiritual care professionals (Nissen et al. 2021). It is recognized, however, that spiritual care remains the most neglected component of palliative care (Gijsberts et al. 2019). A terminal illness causes physical and spiritual suffering, resulting in a greater tendency to question the meaning of life and death (Quinn and Connolly 2023). It has been reported that almost half of the patients in the palliative care services experience spiritual distress (Hui et al. 2010). When patients are diagnosed with terminal illness and possibly facing premature death, increased spiritual distress may contribute to feelings of anxiety, futility, meaninglessness, and a sense of loss (Chan et al. 2014; Goerling et al. 2014; Gonçalves et al. 2023), and people with advanced illnesses express their desire for healthcare professionals to recognize and address their spiritual needs. People with ALS (PALS) can turn to spirituality to manage their disease progression, minimize their suffering, increase their hope, and improve their quality of life. At this point, only a few studies have evaluated the role of spirituality and the role of spiritually integrated interventions for PALS, and most studies are based on qualitative methods to explore spiritual experiences and perceptions (Gonçalves et al. 2023).

Some PALS can find new meanings in the illness situation, turning adversity into an advantage, living and enjoying life to the fullest (Ozanne et al. 2013; Rosengren et al. 2014; Yuan et al. 2021), in a change of perspective, appreciating time and relationships, namely the support of family, friends, and the community (Madsen et al. 2019; Yuan et al. 2021). The consensus among studies indicates, however, that additional research is needed to obtain a comprehensive appreciation of the lived experiences of PALS during the various stages of ALS progression (Gonçalves et al. 2023) and the need for integrative care that includes spiritual needs in which professionals consider this dimension when providing care, encouraging it as a fundamental part of palliative care (Gonçalves et al. 2023; Jaman-Mewes et al. 2024).

Despite the undeniable importance of addressing the spiritual distress and needs of patients with advanced illness, these aspects are often overlooked, resulting in inadequate spiritual care for those facing terminal illness. Diseases such as ALS are even more challenging due to their wide functional and phenotypic variability and unpredictable course. This can lead to significant uncertainties in the spiritual journey of PALS, particularly as they face increasing dependency and the impact of the loss of physical abilities. These changes affect not only their physical well-being but also their deep-seated spiritual domain. The present study aims to identify the spiritual needs and resources of PALS as part of the spiritual care process, to explore how different stages of disease progression and functional profiles affect and correlate with spirituality domains, and to characterize the experiences of PALS in their current palliative care situation.

## Methods

### Study design

**Figure 1.**
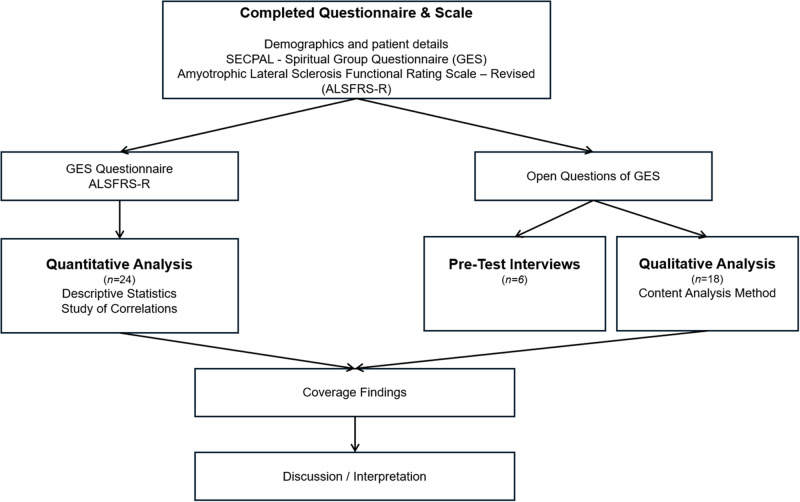
Convergent mixed-methods design schematic.

A convergent mixed methods design was used, supporting multiple approaches to collect and analyze data (Creswell and Creswell 2018) aligned with the study aims. Quantitative and qualitative data were collected concurrently through different instruments (questionnaires and scales). Findings were separately analyzed and later converged and integrated through a side-by-side comparison, allowing for a more comprehensive understanding by complementing and validating both data sources. The conventional content analysis was applied to interpret qualitative data (Bengtsson 2016), complemented by a quantitative approach focused on analyzing the data collected from PALS to comprehensively explore spiritual resources and needs. The design schematic is presented in Fig. 1.

### Participants and data collection

During February 2023, any person of legal age (18 years or older) with a confirmed diagnosis of ALS according to the revised El Escorial revised criteria (Hardiman et al. 2017), who attended the Portuguese Amyotrophic Lateral Sclerosis Association (APELA), was considered eligible, regardless of the stage of the disease, and was invited to participate voluntarily, with no institutional obligation of pressure to do so. Exclusion criteria included: PALS with severe secondary comorbidities including significant cognitive and/or behavioral impairment, frontotemporal dementia, or who were unable to understand subjective measures calculated with the Portuguese version of the Short Portable Mental Status Questionnaire (SPMSQ – normal Pfeiffer) (Rodrigues RMC 2008) and who, therefore, were not able to understand the nature of the study and/or give their consent; and PALS who were not aware of their diagnosis and prognosis and/or have expressed, at least occasionally, their intuition about the possibility of dying (score < 3 according to the Ellershaw Scale) (Ellershaw et al. 1995).

Participants’ baseline sociodemographic and clinical characteristics were collected through a questionnaire and consultation of the clinical file. Pseudonymization was implemented by replacing and coding all identifiable information to protect participants’ identities and guarantee confidentiality. Functionality was assessed using the ALS Functional Rating Scale-Revised (ALSFRS-R), a 12-question instrument designed for monitoring the progression of functional disability in patients with ALS. Four subscores, with 3 questions each, are evaluated: bulbar function (BS), upper limb function (ULS), lower limb function (LLS), and respiratory function (RS). Total ALSFRS-R score ranges from 0 to 48, resulting from the sum of the 4 subgroups, where higher scores indicate higher functionality (Cedarbaum et al. 1999; Gonçalves and Magalhães 2022). The Portuguese version of the GES Questionnaire (developed by Grupo de Espiritualidad de la SECPAL) was used to assess participants’ spiritual resources and needs (Manso et al. 2020). It starts with 6 optional, open-ended questions to create a space of intimacy and a climate of serenity, allowing the exploration of the person’s inner world in a guided way. Followed by 8 items assessing spirituality as a general factor and the 3 spirituality dimension, which aim to assess the needs and, at the same time, experiences and resources of the person through three spiritual dimensions: intrapersonal (need for meaning and coherence), interpersonal (harmony in relationships with significant people and the need to be loved and to love), and transpersonal (need to have hope and leave a legacy that will last beyond himself). The open questions were designed to create and share a space of intimate communication with the patient and establish a climate of trust. The other 8 items were answered on a 5-point Likert-type scale, with responses ranging from 0 (not at all) to 4 (a lot), with a Cronbach’s alpha of 0.72 for the original version (Benito et al. 2014) and 0.65 for the Portuguese version (Manso DMC 2021).

## Study procedure and data analysis

### Qualitative phase

All interviews were conducted, carried out by a trained researcher, and video-recorded. The video recording made it possible to transcribe and code all the topics covered afterward, before their elimination. Although priority was given to conducting interviews in person, a flexible online alternative was offered to conduct the interviews through Zoom^®^ meetings, aiming to prioritize patients’ comfort and consider all functional impairments and transportation challenges. This strategy also allowed patients to minimize and better manage their fatigue during the interviews, as well as for participants with changes in the language and communication process to be able to better manage the interview and communication times with the researcher. Both strategies ensured a calm and comfortable environment, guaranteeing data confidentiality and privacy. The presence of a caregiver or formal caregiver was also allowed upon request, but they could not intervene in the interview.

At the beginning of the interview, it was explained to all participants what spirituality is and its dimensions and that it is not just about religion but about everything that gives meaning to life. A presentation of the GES Questionnaire and its purpose was also made, highlighting that the answers to this questionnaire must be focused on the spiritual dimension of life, that there are no correct or incorrect answers, and that what was intended was to explore concerns and abilities that can affect anyone throughout their life and, in this way, the important thing was that the response was faithful to what they really felt and experienced (Benito et al. 2014; Manso et al. 2020). Finally, space was always open for doubts and clarifications if needed.

The researcher received training to conduct the interviews by undergoing a pre-test in which the GES Questionnaire was applied to 6 patients with ALS who did not participate in the qualitative phase of the study. This approach validated the ideal conditions for collecting information regarding the expected duration of the interview, the patient’s ways of communication, their communication pre-requirements, and symptom management such as fatigue (Fig. 1).

The qualitative analysis of the data obtained was based on the Content Analysis Method, respecting the sequential steps: pre-analysis, exploration of the material and treatment of results, and inference and interpretation of results (Bengtsson 2016), using ATLAS.ti^®^ software version 8.4.5 for data analysis. Data were coded (inductive descriptive labels); irrelevant information was discarded and then categorized through a comparative and iterative analytic process. The sample size for qualitative data was defined as 18 participants, according to the thematic saturation point (Hennink and Kaiser 2022). Validity was increased through triangulation of qualitative and quantitative data. Quantitative and qualitative datasets were integrated during data analysis.

### Quantitative phase

Quantitative analysis was carried out using the SPSS^®^ software version 29.0 for Windows^®^ from IBM^®^. Descriptive statistics were used: frequency and measures of central tendency and dispersion to characterize the sample. A significance level of 0.05 was considered. The Cronbach’s alpha (to assess internal consistency) and Spearman or Pearson coefficients were used to study correlations between variables without or with normal distribution, respectively, according to the normality of the distribution, assessed by applying the Shapiro−Wilk test and Fisher’s exact test. Finally, the Student’s *t*-parametric test was used for independent samples, when comparing different groups, after ensuring a normal distribution.

## Results

In this study, of the 25 patients contacted, 24 were eligible to participate in the quantitative phase and 18 in the qualitative phase, from which 13 underwent presential interviews and 5 online ones. The reason to exclude one contacted patient was a slight deterioration in the ability to understand subjective measures.

### Quantitative results

Of the 24 participants, 54.2% were male (*n* = 13 males). The mean age of the sample was 57.6 (±14.2) years, with an ALSFR-R total score mean value of 24.83 (±8.69) and a disease onset of 4.8 (±3.1) years. Table 1 shows data related to the participants’ sociodemographic characterization and disease-related aspects.

**Table 1.** Characterization of participants’ sociodemographic and disease-related aspects of the participants

	All group	Qualitative analysis group
**Sociodemographic and clinical characteristics**	**(*n* = 24)**	**(*n* = 18)**
	**x̅ (min/max) ± SD**	**x̅ (min/max) ± SD**
**Age (in years)**	57.6 (±14.2)	59.5 (32/80) ± 14.7
**Gender**	***n* (%)**	***n* (%)**
Male	13 (54.2)	10 (55.6)
Female	11 (45.8)	8 (44.4)
**Education Level**	***n* (%)**	***n* (%)**
Basic Education	2 (8.3)	1 (5.6)
Lower Secondary Education	3 (20.8)	3 (16.7)
Upper Secondary Education	4 (25.0)	4 (22.2)
Bachelor/Master	7 (33.3)	7 (38.9)
PhD	3 (12.5)	3 (16.7)
**Communication**	***n* (%)**	***n* (%)**
Speech	17 (70.8)	14 (77.8)
Direct Augmentative System (via eye tracker)	7 (29.2%)	4 (22.2)
**Informal Caregiver’s Relationship Level**	***n* (%)**	***n* (%)**
Spouse	17 (70.8%)	13 (72.2%)
Mother	3 (12.5%)	2 (11.1%)
Girlfriend	2 (8.3%)	1 (5.6%)
Niece	1 (4.2%)	1 (5.6%)
Daughter	1 (4.2%)	1 (5.6%)
**Formal Caregiver’s Support**	***n* (%)**	***n* (%)**
Yes vs. No	15 (62.5) vs. 9 (37.5)	13 (72.2) vs. 5 (27.8)
	**x̅ (min/max) ± SD**	**x̅ (min/max) ± SD**
**Symptoms Onset (in years)**	4.8 (±3.1)	5.7 (1/12) ± 2.9
***ALSFRS-R* Scale**	24.83 (±8.69)	21.28 (3/37) ± 9.86

Regarding the GES Questionnaire, the Cronbach’s alpha was calculated (0.73) to assess the scale’s internal consistency. This result is in line with the reliability indices reported for both the original version and the Portuguese adaptation of the instrument, presenting an adequate level of internal consistency, supporting the reliability of the instrument in our sample, and reinforcing the scale’s stability in measuring the intended construct in our sample size (*n* = 24).

The responses to the GES Questionnaire statements revealed a strong spiritual well-being among the participants, with a high percentage identifying “Very” or “A lot” with most statements (ranging from 77.8% to 100%). The only exception was the 8^th^ statement, “*I feel connected to a supreme reality (o supreme Being, nature, God, …)*,” where the majority (55.5%) identified “Not much” or “Not at all.” The 4^th^ question, “*I feel loved by the people who are important to me*,” which falls under the Interpersonal spiritual domain, received 100% of ‘Very’ or “A lot” responses.

Applying the *t-*test for independent samples, a statistically significant difference (mean ± SD) was identified for the Intrapersonal domain between males (8.90 ± 2.60) and females (11.13 ± 1.13) (*p* = 0.035). No differences were identified between the Interpersonal and Transpersonal domains or the total score of the instrument applied and the participants’ gender. A moderate positive correlation was found between the age of the participants and the sum of the statements in the GES Questionnaire (*r* = 0.556; *p* = 0.017) and between the Intrapersonal domain of the GES Questionnaire and age (*r* = 0.576; *p* = 0.012) but not for the Interpersonal and Transpersonal domains. In particular, the first statement in the questionnaire, “*Looking back on my life, I feel satisfied with what I have lived and with myself*,” is positively correlated with age (ρ = 0.635, *p* = 0.022) and the second statement, “*I’ve done (accomplished) in my life what I felt I had to do*” (ρ = 0.682, *p* = 0.011).

A negative correlation was identified between the 8^th^ statement, “*I feel connected to a supreme reality* (..)” and the level of educational qualifications (*r* = −0.36; *p* = 0.0722). No significant correlations were found between the functional scale (ALSFRS-R total score or subdomains) and the spiritual dimensions.

### Qualitative analysis

The 18 participants interviewed were between 32 and 80 years old, and 55.6% were male. Regarding educational qualifications, most of the sample had a high level of literacy (bachelor’s degree and doctorate). Regarding aspects of the illness, the majority, 77.8%, use speech to communicate intelligibly (despite some having detectable speech disturbances). Spouses represented 72.2% (*n* = 13) of the informal caregivers. Most participants, 72.2%, also have the support of a formal caregiver. The duration of the illness ranged from 1 year to 12 years, and participants were at different stages of functional dependence (Table 1).

## Initial open questions: ALS patients’ perception of their inner world

Analyzing the main topic of the initial open questions of the GES questionnaire, 6 categories were established related to the inner world of PALS at different stages of the disease’s progression: *Concern, Nuisance, Help, Support, Safety*, and *Valorization*. These categories encompass 23 subcategories that refer to aspects that are part of the interviewees’ experience in the process of a terminal illness. Table 2 provides a detailed overview of all the categories and subcategories.

**Table 2.** Overview of categories and subcategories

Categories	**Subcategories** Ex. “support citations,” In	Interviews that contributed to the results *n* (%)	Number of quotations
**Concern**	**Future**		
	“(..) *another thing that worries me is suffering* (..)” I11	I1, I4, I5, I6, I7, I8, I11, I13, I16	12
	“*To know that there is no euthanasia in case I am suffering a lot*.” I13	*n* = 9 (50%)	
	“*The family in general because I know that no one misses anyone, but I know I will miss my family.*” I7		
	**Functional and symptomatologic impact of the disease**		
	“*At the moment, what worries me most is depending on others. I can’t accept help because I have always been the one who helped others, and now it hurts me that others take care of me. And that worries me because I know what it’s like. After all, I’ve been there.*” I2	I2, I3, I9, I15, I18	7
	“*Not having peace with the difficulties that the disease imposes. I’m tired, and I’ve accepted everything; I’ve done what I can, but now I just need peace.*” I15	*n* *=* *5* (28%)	
	**Impact of the disease on the family**		
	*“Look, it is me and my sister who are semi-terminally ill and how are other people going to adapt to that.*” I14	I3, I8, I9, I10, I11, I12, I14, I16, I17	12
	“*The work I give to the family* (..)” I10	*n* *=* 9 (50%)	
	*“My family, because I am in the state that I am, I feel like they are in deep suffering.*” I12		
**Nuisance**	**Functional and symptomatologic impact of the disease**		
	“*Physically, the difficulties of the disease, breathing, pain, constipation.*” I15	I3, I2, I8, I9, I10, I12, I13, I15, I18	12
	*“Being dependent on others because I have always been a person who helped others, and now I have to be helped.”* I12	*n* *=* 9 (50%)	
	**Emotional and social problems**		
	“(..) *another thing is relevance; it bothers me that I was a person with some relevance, at the moment, I don’t have it.*” I11	I1, I2, I4, I5, I11, I13, I15, I17	12
	“*I gradually lost purpose, stopped going out to socialize and stopped doing other activities; I just no longer do.*” I17	*n* = 8 (44%)	
	**Organizational problems**		
	“(..) *everything takes place within deadlines that are not compatible with the disease, especially those with a very rapid progression. This inefficiency and unnecessary obstacles bothers me.*” I16	I6, I14, I16 *n* = 3 (17%)	3
	**Uncertainties about the future**		
	*“I know what I’m going through now, but I don’t know what’s in store for me at the end of my life.”* I7	I7, I8, I14	5
	“*If I’m going to stop talking* (..) *and unable to express myself in what I think I should, that worries me.*” I8	*n* = 3 (17%)	
**Help**	**Coping strategies**		
	“*Not thinking. It looks like I have an on-and-off button. I completely shut down and don’t think.*” I1	I1, I3, I4, I5, I6, I9, I10, I13, I14, I15, I16	18
	*“It’s the use of work that still makes me feel active.* (..) *it gives me a sense of reality that I’m still alive; I’m not just surviving.*” I6	*n* = 11 (61%)	
	**Informal social interaction**		
	“*It’s people’s affections and the affection they give me which brings joy. It’s like you are taking another step forward in life.*” I3	I3, I7, I10, I11, I14, I15, I18 *n* = 7 (39%)	8
	**Transcendent**		
	“*When I’m down, I pray. Pray for you and my friends (…). I feel like it helps me on a spiritual level and on a religious level. Otherwise, you get into a cycle that doesn’t work, and I was falling for it.”* I1	I7, I8, I12 *n* = 3 (17%)	4
	**Community support**		
	“*What helps me most is the electric wheelchair because it gives me independence, because I always been very free*” I2	I2, I7, I9, I17	4
	“(..) *is to come to APELA* (..)” I7.	*n* = 4 (22%)	
	“(..) *having a personal assistant.*” I9		
**Support**	**Formal social interaction**		
	“(..) *talk to someone who makes me want to expose myself* (..) *with the psychologist. The time I’ve been with her has already created an opening in me that I never allowed before.*” I6	I6, I9, I16 *n* = 3 (17%)	3
	**Informal social interaction**		
	“*My daughter and, oddly enough, my granddaughter is always there. Even my son-in-law helps. They are my family*.” I5	I1, I2, I3, I5, I7, I8, I9, I10, I11, I12, I13, I14, I15, I16, I17, I18	20
	“*Usually my wife, partner of almost 40* *years.*” I17	*n* = 16 (89%)	
	**Transcendent**		
	“*Now more than ever I turn to Our Lady of Fátima. Before I had the illness, I went to Fátima, and there was something different that touched me a lot.* (..) *sometimes I pray with her*.” I8	I8 *n* = 1 (6%)	3
	**Self-support**		
	“*I find refuge in things like music, reading* (..)” I4	I4, I15, I16	4
	“*I would say that the first person is myself; I think that if I don’t find a way to deal with things, it’s unlikely that anyone else will solve my problems or will be able to help me in a way that works for me.*” I16	*n* = 3 (17%)	
	**Absence of crisis situations**		
	“*I don’t have crises, neither do I have distress in my life, thank God.*” I18	I18 *n* = 1 (6%)	1
**Safety**	**Coping strategies**		
	“*I turn to thoughts* (..) *I no longer think about the next day; I think about the day I live in.*” I6	I6, I8, I15, I16	6
	“(..) *I don’t like to be supported on the street by others, so I try no to give the image that I’m bad* (..) *Sometimes, going down the stairs, neighbours come there, and I stop, I disguise, and then I continue.*” I8	*n* = 4 (22%)	
	**Support network**		
	“*The public structure, the patients” association, physiotherapy, hospitals work. I know that if I have a problem, I have somewhere to turn*.” I14	I4, I6, I14 *n* = 3 (17%)	3
	**Informal social interaction**		
	“*The person I feel safe with is my wife. I know that she understands anything. She almost always knows what I need, without having to ask for anything*.” I1	I1, I2, I3, I4, I5, I8, I9, I10, I12, I14 *n* = 10 (56%)	12
	**Transcendent**		
	“*For example, with the help I received in the past with Reiki, I finished the sessions and felt free.* (..) *I also have prayer and meditation*.” I6’	I6, I13, I18	6
	“*I know that I have never been alone because I always feel good presences that warn me through dreams.*” I13	*n* = 3 (17%)	
	“*God helps me to have strength and I ask God a lot to help me*.” I18		
	**Lack of safety**		
	“*I no longer feel safe because it no longer depends solely on me. The feeling if being safe is over*.” I12	I6, I11, I12, I13, I15, I17	11
	“(..) *mentally it is another matter, and it is already irremediable.*” I15	*n* = 6 (33%)	
**Valorization**	**Personality characteristics**		
	*“(..) the trust and earnestness they placed in me*.” I3	I3, I5, I7, I8, I9, I10, I11, I12, I13, I14, I15, I16, I17, I18	25
	“*Can I say my strength? They say that I am a person with a lot of strength to go through what I have* (..) *that I will not let myself go down*.” I7	*n* = 14 (78%)	
	**Social and family role**		
	“*I’ve been told several times that I always think about others first*.” I1	I1, I2, I3, I4, I6, I8	6
	“*At home, my opinions always prevail. I feel like I’m the matriarch of the family*.” I2	*n* = 6 (33%)	

## Categories

*Concern (Future)*: half of the sample talked about the future, expressing concerns regarding choices that might need to be taken in the future, suffering, and the impacts of this journey on the family and loved ones.

*Concern and Nuisance (Functional and Symptomatologic Impact of the Disease)*: the functional impact of ALS and patients’ symptoms is also a highlighted concern and was mentioned by 50% of the participants in the *Nuisance* category and by 28% in the *Concern* category, especially considering the balance between increasing dependency and acceptance of help.

*Help and Safety (Coping Strategies)*: coping strategies are common in both the *Help* and *Safety* categories, with a greater weight in Help (61%). These are a positive outlook and dedication to hobbies, maintaining normality, avoiding confrontation and constant thoughts about the illness, and anticipating and coping with difficulties, while looking to sustain self-preservation, a sense of reality, and self-utility.

*Help, Support, and Safety*: regarding these categories, two subcategories emerged in common.

*Informal Social Interaction*: family plays a crucial role in practical and emotional matters, but it is also a source of purpose and protection. The spouse plays a unique and fundamental role and is the family member highlighted in all three categories by 50% of the participants. Despite being mentioned in Help and Support, friends are not valued as a security source.

*Transcendent*: faith and prayer appear as sources of connection with a higher power in 22% of the participants. In the *Support* and *Safety* categories, other strategies like Mindfulness, Reiki, and Meditation were mentioned by 11% of the participants.

Valorization: regarding this category, participants had some difficulty pointing out aspects that the most significant ones might value in themselves, and in the reflection process, they frequently highlighted self-resiliency, trustworthiness, and social and family roles.

## Discussion

### Concern (future)

In the Concern category, half of the sample talked about the future. In a situation of illness, there is no suffering without envisioning the future and anticipating what may come for oneself and one’s family, so the possibility that there may be nothing to end or alleviate suffering is a concern (Krikorian and Limonero 2012). On the other hand, anticipation and prevention can be important in the search for normality (Brown and Addington-Hall 2008).

### Concern and nuisance (functional and symptomatologic impact of the disease)

The functional and symptomatic impact of the disease is mentioned by 50% of the participants in the *Nuisance* category and by 28% in the Concern category. In both cases, the loss of autonomy and the need to accept dependence seem to be demanding, given the burden PALS place on caregivers and their desire to maintain normality. It is uncomfortable for them to perceive that they are witnessing their functional decline without being able to do anything about it as new symptoms appear (Yuan et al. 2021) and day-to-day reality shifts away from normative performances (Ng et al. 2017). At a more advanced stage of the disease, the concern is about extreme fatigue, which impacts relationships and leads to a progressive loss of purpose, as also previous highlighted by different investigators (Hui et al. 2010; Quinn and Connolly 2023).

### Help and safety (coping strategies)

Coping strategies are common in both the *Help* and *Safety* categories, with a greater weight in Help (61%). These are a positive outlook and dedication to hobbies, maintaining normality, avoiding confrontation and constant thoughts about the illness, anticipating and coping with difficulties, having a prepared support network, and hoping for the possibility of an innovative drug (Gonçalves et al. 2023; Hamama-Raz et al. 2021; Leandro et al. 2022).

### Help, support, and safety

Regarding these categories, two subcategories emerged in common.

### Informal social interaction

Family plays a crucial role in practical and emotional matters, but it is also a source of purpose and protection (Foley et al. 2007; Yuan et al. 2021). The spouse plays a unique and fundamental role and is the family member highlighted in all three categories by 50% of the participants. Despite being mentioned in Help and Support, friends are not valued as a security source, which may explain the avoidance and social isolation mentioned (Sommers-Spijkerman et al. 2023).

### Transcendent

The second subcategory in common is the Transcendent. Faith and prayer appear as sources of connection with a higher power in 22% of the participants. In the *Support* and *Safety* categories, 11% mentioned Reiki, Mindfulness, and Meditation, but there are few studies that address, adapt, and test these therapies on PALS. It should be noted that, unlike other studies, this category was the least relevant (O’Brien and Clark 2015; Pagnini et al. 2013).

### Valorization

Finally, in the *Valorization* category, the participants had some difficulty pointing out aspects that the most significant ones might value in themselves, and it turned out to be a very interesting exercise in reflection. Personality traits and social and family roles come up. PALS appreciate feeling recognized and valuable to others, as it helps them find meaning (Budych et al. 2012; Hamama-Raz et al. 2021; Ozanne et al. 2013).

Contrary to what was hypothesized in our study, spiritual needs and resources did not seem to vary with the functional status of the PALS. As expected, spiritual needs and resources tend to vary with age, with younger ages presenting a more fragile spiritual dimension overall. Also, the interpersonal and spiritual dimension seems to play a central role in the lives of PALS. In this study, the Transpersonal spiritual dimension is scored less valued among the participants, unlike other studies in which participants report the importance of the transcendent (Fanos et al. 2008; O’Brien and Clark 2015). Also, people with higher educational attainment are more rational and practical, and some have never felt any connection with the Transpersonal dimension, even before the illness. This relationship has not yet been explored in other studies, so it will be essential to address it in future research. Considering the current perspective on spirituality (Nolan et al. 2011) and the experiences of PALS, we propose that the relationship with oneself and that with others are the most crucial spiritual components in the quest for life’s meaning, in line with previous suggestions (Gonçalves et al. 2023; Nolan et al. 2011), especially considering the rapid progression of the disease and the subsequent need for adjustment due to cumulative functional losses. Notably, the significance of the Interpersonal dimension in our study and others may be linked to the substantial role we assign to family and interpersonal relationships in the Mediterranean area, a region known for its strong family ties and social support systems (Galiana et al. 2014).

Female participants seem to feel more fulfilled and well-resolved in the Intrapersonal spiritual domain compared to males. A previous study also shows the resilience of self-management in the spiritual domain of women with ALS (Rosengren et al. 2014). Regarding age, it was previously reported in patients with advanced cancer that for young patients, the possibility of a limited life of unfulfilled aspirations can be a source of great spiritual and psychosocial suffering (Hui et al. 2010). The same conclusions were reached in Chochinov’s study of patients receiving palliative care (Chochinov et al. 2009). Our results are suggestive of the same spiritual challenges regarding younger adults with ALS, especially considering the lower contribution of the intrapersonal expectations to the overall spiritual and psychological experiences.

The qualitative analyses elucidated the process of change in the participants’ perception of life, providing the opportunity to talk about their spiritual needs and to target possible existing spiritual resources, which are essential in the individualization and planning of spiritual care. When considering the spiritual resources and needs, which are still within the qualitative component of the study, it is clear that some participants made free-form reflections during the exposition of the statements in the GES Questionnaire. In this study, anticipation regarding the future dominated the concerns expressed by the participants. In a situation of illness, there is no suffering without envisioning the future and anticipating what may come for oneself and one’s family, so the possibility that there may be nothing to end or alleviate suffering is a concern (Krikorian and Limonero 2012). On the other hand, anticipation and prevention can be important in the search for normality. The loss of autonomy and the need to accept dependence seem to be demanding, given the burden PALS places on caregivers and their desire to maintain normality as day-to-day reality shifts away from normative performances. It is uncomfortable for them to perceive that they are witnessing their functional decline without being able to do anything about it as new symptoms appear (Yuan et al. 2021). At a more advanced stage of the disease, the concern is about extreme fatigue, which impacts relationships and leads to a progressive loss of purpose, as also previously highlighted by different investigators (Ng et al. 2017; Quinn and Connolly 2023). Coping strategies represent a valuable resource to help and support PALS, playing a crucial role in the adjustment during the disease trajectory, which includes keeping the individual active and securing a safe with a body and mind fulfilling routine, avoiding conflicts and persistent negative thoughts, but being able to anticipate and to cope with difficulties, having a prepared support network, and hoping for the possibility of innovative drugs (Gonçalves et al. 2023; Hamama-Raz et al. 2021; Leandro et al. 2022).

The resources transmitted in the Intrapersonal domain are related to the feeling of accomplishment and inner peace resulting from the sensation of having lived fully (Rosengren et al. 2014). In our results, personality traits and social and family roles frequently appeared in the valuation analysis, prompting reflection on the question, “What do people value about you?” PALS appreciate feeling recognized and valuable to others, as it helps them find meaning. The courage, love, and personal development acquired throughout life strengthen the relationship with oneself and the meaning of life (Foley et al. 2007; Locock et al. 2009). Other PALS reported feeling needs such as better managing the balance between work and enjoying life, and for others who feel disconnected and lost, the search for meaning in life is highlighted (Ozanne et al. 2013). In the interpersonal spiritual domain, the results of our study are aligned with previous works where the vast majority of PALS turned to family and friends for support, highlighting the crucial role of these relationships in their spiritual well-being (Fanos et al. 2008; Murphy et al. 2009). However, our results stress that despite representing a source of help and support, friends are not perceived and valued as a security source, which may explain the avoidance and social isolation mentioned (Sommers-Spijkerman et al. 2023). Many participants feel at peace and reconciled with the people around them because they know how to ask for forgiveness. In addition, altruism and passing on these and other values to the next generation strengthen the relationships and usefulness of the interviewees (Yuan et al. 2021). The needs in this domain are related to resolving some family conflicts, feeling useful despite functional limitations, and especially caring for those who care for them (Foley et al. 2007; Rosengren et al. 2014). In the Transpersonal spiritual domain, they refer to help from higher entities, from nature and the universe, or from their closest ancestors (O’Brien and Clark 2015). Others believe that practicality and rationality are the best resources (Fanos et al. 2008; Hamama-Raz et al. 2021). It should be noted that, unlike other studies (O’Brien and Clark 2015; Pagnini et al. 2013), the transcendental category, qualitative phase, was the least relevant. Moreover, they also mention hope, and the needs expressed in this domain are fewer than the previous ones. Some participants say they have lost their purpose in life, and all hope that a drug or some cure for their illness will be found in time. This highlights the double side of hope, where for some PALS, it represents an obstacle to achieving control and actively coping with the disease, and to other PALS, hope is a crucial resource of empowerment, coping, and control (Hamama-Raz et al. 2021).

Our work also emphasizes the benefit of the “guided conversation” during the questionnaire, which facilitates the dialogue and leads to a valuable and self-perceived reflection by the PALS, particularly about valorization aspects that matter to them, the meaning of life and their journey. These conversations are assisted through spiritual needs and spiritual assessment tools and are also useful as therapeutical approaches (Ichihara et al. 2019; Jaman-Mewes et al. 2024); furthermore, it can be intertwined with unstructured complementary conversations.

**Figure 2.**
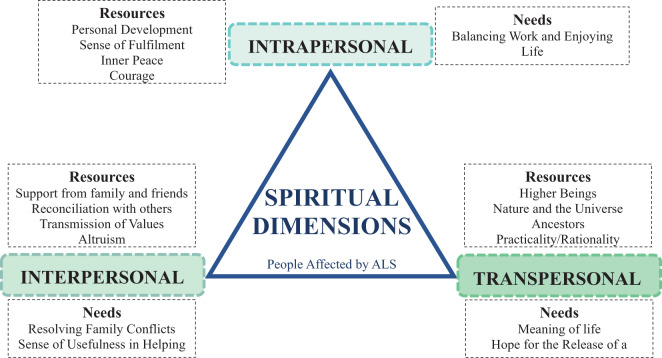
Spiritual needs and resources of people affected by amyotrophic lateral sclerosis (ALS), based on the GES questionnaire.

The diagram of spiritual needs and resources (Fig. 2) emerges from the discussion of the results of the statements in the GES Questionnaire.

Spirituality impacts the patients’ disease journey and is a critical component that can impact the suffering, especially existential suffering. When properly addressed, it has the potential to lead to higher levels of spiritual well-being and quality of life and lower levels of uncertainty and conflict regarding decision-making in health. Our findings suggest that delaying the incorporation of the spiritual domain as the disease advances may not be advisable. Instead, we advocate for an early integration of spirituality, starting from the diagnosis by a trained multidisciplinary team in ALS clinics or similar settings. This approach could enable patients to delve deeper into their spiritual domains and potentially mitigate the intensity of future spiritual or existential suffering (Aoun et al. 2016, 2017). Recognizing spiritual needs and working on them promptly are the needs that healthcare providers and caregivers should share (Corpuz 2024), helping PALS to face different challenges of spiritual suffering on the path to spiritual integrity through addressing these issues and the humanization of care. Recognizing and working around known barriers to providing holistic care that addresses spirituality and focuses on an advanced plan of care, such as the lack of opportunity to have conversations about these topics, reluctance to discuss death and dying, concerns about the impact of advanced care plan in family dynamics, and time constraints for professionals to dedicate to the global care, with the articulation of strategies in an intra- and transdisciplinary context are essential.

This study has some potential limitations. First, despite being a rare disease, the present study includes a small sample for the quantitative part. Second, all the samples had the support of a multidisciplinary team, and a considerable part of the PALS have access to formal caregivers or personal assistants, which may, in some way, not correspond to the reality of PALS in comparison to other areas. The presence versus absence of this support might impact patients’ global needs and, in particular, their spiritual needs. We also have to consider the interviewer’s bias and the social desirability of the answers; we have tried to control and minimize it with the pre-case study and the formal script that was followed by the interviewer with the same criteria using the exact words. Furthermore, the participants already knew the researcher, so they felt more comfortable approaching the topics and exploring their dimensions.

We suggest that future studies should explore this topic at different time points of patients’ disease trajectory (longitudinal evaluation), in order to compare its progress and eventual changes. In addition, future research is needed to assess optimal strategies in clinical and homecare settings that address spiritual care, resources, and needs. It is also important to recognize the need for greater investment in health professionals’ education, preparation, and training to use tools to guide them in addressing the spiritual dimension in all trajectories. Encouraging health professionals, patients, family members, and the community to take this dimension into account as a valuable component in palliative care, in health in general, and everyday life, from the early phase of the disease, independently of functional progression rates or functional decline.

## Conclusions

The results of this study reinforce the importance of individualized, comprehensive, and sensitive approaches in care and support practices for PALS, which should include spiritual care regardless of the functional state and capability of the person suffering from ALS. Our results also highlight the special attention that should be given to the intrapersonal and interpersonal domains, assessing needs and resources from an early phase. The fact that experiences in the context of illness, spiritual needs, and resources is essential to consider in a holistic approach to health. Assessing the different dimensions of spirituality and establishing an intradisciplinary plan of care with a structured intervention plan aimed at spiritual intervention in PALS and their caregivers, helping and guiding them in their needs, and considering the resources that matter to them.

## References

[ref1] Aoun SM, Breen LJ, Howting D, et al. (2016) Receiving the news of a diagnosis of motor neuron disease: What does it take to make it better? *Amyotrophic Lateral Sclerosis and Frontotemporal Degeneration* 17(3-4), 168–178. doi:10.3109/21678421.2015.1111907 26609553

[ref2] Aoun SM, Breen LJ, Oliver D, et al. (2017) Family carers’ experiences of receiving the news of a diagnosis of motor neurone disease: A national survey. *Journal of the Neurological Sciences* 372, 144–151. doi:10.1016/j.jns.2016.11.043 28017202

[ref3] Bengtsson M (2016) How to plan and perform a qualitative study using content analysis. *NursingPlus Open* 2, 8–14. doi:10.1016/j.npls.2016.01.001

[ref4] Benito E, Oliver A, Galiana L, et al. (2014) Development and validation of a new tool for the assessment and spiritual care of palliative care patients. *Journal of Pain and Symptom Management* 47(6), 1008–1018.e1001. doi:10.1016/j.jpainsymman.2013.06.018 24099897

[ref5] Brown J and Addington-Hall J (2008) How people with motor neurone disease talk about living with their illness: A narrative study. *Journal of Advanced Nursing* 62(2), 200–208. doi:10.1111/j.1365-2648.2007.04588.x 18394032

[ref6] Bryson KA (2004) Spirituality, meaning, and transcendence. *Palliative and Supportive Care* 2(3), 321–328. doi:10.1017/S1478951504040428 16594418

[ref7] Budych K, Helms TM and Schultz C (2012) How do patients with rare diseases experience the medical encounter? Exploring role behavior and its impact on patient–physician interaction. *Health Policy* 105(2), 154–164. doi:10.1016/j.healthpol.2012.02.018 22464590

[ref8] Cedarbaum JM, Stambler N, Malta E, et al. (1999) The ALSFRS-R: A revised ALS functional rating scale that incorporates assessments of respiratory function. BDNF ALS Study Group (Phase III). *Journal of the Neurological Science* 169(1-2), 13–21. doi:10.1016/s0022-510x(99)00210-5 10540002

[ref9] Chan CW, Choi KC, Chien WT, et al. (2014) Health-related quality-of-life and psychological distress of young adult survivors of childhood cancer in Hong Kong. *Psychooncology* 23(2), 229–236. doi:10.1002/pon.3396 24027211

[ref10] Chochinov HM, Hassard T, McClement S, et al. (2009) The landscape of distress in the terminally ill. *Journal of Pain and Symptom Management* 38(5), 641–649. doi:10.1016/j.jpainsymman.2009.04.021 19713069

[ref11] Corpuz JCG (2024) Integrating spirituality in the context of palliative and supportive care: The care for the whole person. *Palliative and Supportive Care* 22(2), 408–409. doi:10.1017/S1478951523001268 37606048

[ref12] Creswell JW and Creswell JD (2018) *Research Design: Qualitative, Quantitative, and Mixed Methods Approaches, LA*, 5th edn. Los Angeles: SAGE Publications.

[ref13] Ellershaw JE, Peat SJ and Boys LC (1995) Assessing the effectiveness of a hospital palliative care team. *Palliative Medicine* 9(2), 145–152. doi:10.1177/026921639500900205 7541684

[ref14] Fanos JH, Gelinas DF, Foster RS, et al. (2008) Hope in palliative care: From narcissism to self-transcendence in amyotrophic lateral sclerosis. *Journal of Palliative Medicine* 11(3), 470–475. doi:10.1089/jpm.2007.0098 18363490

[ref15] Foley G, O’Mahony P and Hardiman O (2007) Perceptions of quality of life in people with ALS: Effects of coping and health care. *Amyotrophic Lateral Sclerosis: Official Publication of the World Federation of Neurology Research Group on Motor Neuron Diseases* 8(3), 164–169. doi:10.1080/17482960601164532 17538778

[ref16] Galiana L, Oliver A, Gomis C, et al. (2014) Cuestionarios de evaluación e intervención espiritual en cuidados paliativos: una revisión crítica. *Medicina Paliativa* 21(2), 62–74. doi:10.1016/j.medipa.2013.02.003

[ref17] Gijsberts MHE, Liefbroer AI, Otten R, et al. (2019) Spiritual care in palliative care: A systematic review of the recent European literature. *Medical Sciences (Basel)* 7(2), 1–21. doi:10.3390/medsci7020025 PMC6409788 30736416

[ref18] Goerling U, Jaeger C, Walz A, et al. (2014) The efficacy of short-term psycho-oncological interventions for women with gynaecological cancer: A randomized study. *Oncology* 87(2), 114–124. doi:10.1159/000362818 25012072

[ref19] Gonçalves F and Magalhães B (2022) Effects of prolonged interruption of rehabilitation routines in amyotrophic lateral sclerosis patients. *Palliat Support Care* 20(3), 369–374. doi:10.1017/s1478951521000584 33942709

[ref20] Gonçalves F, Teixeira MI and Magalhães B (2023) The role of spirituality in people with amyotrophic lateral sclerosis and their caregivers: Scoping review. *Palliat Support Care* 21(5), 914–924. doi:10.1017/s1478951522001511 36464916

[ref21] Hamama-Raz Y, Norden Y and Buchbinder E (2021) The double sides of hope: The meaning of hope among amyotrophic lateral sclerosis (ALS) patients. *Death Studies* 45(3), 238–247. doi:10.1080/07481187.2019.1626946 31192774

[ref22] Hardiman O, Al-Chalabi A, Chio A, et al. (2017) Amyotrophic lateral sclerosis. *Nature Reviews Disease Primers* 3, 17071. doi:10.1038/nrdp.2017.71 28980624

[ref23] Hennink M and Kaiser BN (2022) Sample sizes for saturation in qualitative research: A systematic review of empirical tests. *Social Science & Medicine* 292, 114523. doi:10.1016/j.socscimed.2021.114523 34785096

[ref24] Hui D, de la Cruz M, Thorney S, et al. (2010) The frequency and correlates of spiritual distress among patients with advanced cancer admitted to an acute palliative care unit. *American Journal of Hospice and Palliative Medicine®* 28(4), 264–270. doi:10.1177/1049909110385917 21057143

[ref25] Ichihara K, Ouchi S, Okayama S, et al. (2019) Effectiveness of spiritual care using spiritual pain assessment sheet for advanced cancer patients: A pilot non-randomized controlled trial. *Palliat Support Care* 17(1), 46–53. doi:10.1017/s1478951518000901 30683167

[ref26] Jaman-Mewes P, Caetano da Silva de Oliveira M, Regina Mazotti M, et al. (2024) Spiritual care interventions for palliative care patients: A scoping review. *Palliative and Supportive Care* 22(5), 1449–1468. doi:10.1017/S1478951524000592 39136153

[ref27] Kiernan MC, Vucic S, Cheah BC, et al. (2011) Amyotrophic lateral sclerosis. *Lancet* 377(9769), 942–955. doi:10.1016/s0140-6736(10)61156-7 21296405

[ref28] Krikorian A and Limonero JT (2012) An integrated view of suffering in palliative care. *Journal of Palliative Care* 28(1), 41–49. doi:10.1177/082585971202800107 22582471

[ref29] Leandro GS, Dourado Júnior MET, Santana GC, et al. (2022) Coping strategies among amyotrophic lateral sclerosis (ALS) patients: An integrative review. *Journal of Neurology* 269(2), 693–702. doi:10.1007/s00415-021-10472-2 33783642

[ref30] Locock L, Ziebland S and Dumelow C (2009) Biographical disruption, abruption and repair in the context of Motor Neurone Disease. *Sociology of Health and Illness* 31(7), 1043–1058. doi:10.1111/j.1467-9566.2009.01176.x 19659736

[ref31] Madsen LS, Jeppesen J and Handberg C (2019) “Understanding my ALS.” Experiences and reflections of persons with amyotrophic lateral sclerosis and relatives on participation in peer group rehabilitation. *Disability & Rehabilitation* 41(12), 1410–1418. doi:10.1080/09638288.2018.1429499 29373921

[ref32] Manso DM, Benito E, Oliver A, et al. (2020) Questionário GES: avaliação de recursos e necessidades espirituais. *Revista Psicologia, Saúde & Doenças* 23(10), 4–9.

[ref33] Manso DMC ML (2021) Tradução e adaptação transcultural para português europeu do Cuestionario GES (SECPAL. *Cadernos de Saúde* 13(2), 21–32. doi:10.34632/cadernosdesaude.2021.9998

[ref34] Moberg DO (2002) Assessing and measuring spirituality: Confronting dilemmas of universal and particular evaluative criteria. *Journal of Adult Development* 9(1), 47–60. doi:10.1023/A:1013877201375

[ref35] Murphy V, Felgoise SH, Walsh SM, et al. (2009) Problem solving skills predict quality of life and psychological morbidity in ALS caregivers. *Amyotrophic Lateral Sclerosis* 10(3), 147–153. doi:10.1080/17482960802245007 18618351

[ref36] Ng L, Khan F, Young CA, et al. (2017) Symptomatic treatments for amyotrophic lateral sclerosis/motor neuron disease. *Cochrane Database SystRev* 1(1), Cd011776. doi:10.1002/14651858.CD011776.pub2 PMC6469543 28072907

[ref37] Nissen RD, Viftrup DT and Hvidt NC (2021) The process of spiritual care. *Frontiers in Psychology* 12, 674453. doi:10.3389/fpsyg.2021.674453 34557128 PMC8453153

[ref38] Nolan S, Saltmarsh P and Leget C (2011) Spiritual care in palliative care: working towards an EAPC task force. *European Public Law* 18(2).

[ref39] O’Brien MR and Clark D (2015) Spirituality and/or religious faith: A means for coping with the effects of amyotrophic lateral sclerosis/motor neuron disease? *Palliative and Supportive Care* 13(6), 1603–1614. doi:10.1017/S1478951515000097 25851240

[ref40] Ozanne AO, Graneheim UH and Strang S (2013) Finding meaning despite anxiety over life and death in amyotrophic lateral sclerosis patients. *Journal of Clinical Nursing* 22(15-16), 2141–2149. doi:10.1111/jocn.12071 23387287

[ref41] Pagnini F, Di Credico C, Gatto R, et al. (2013) Meditation training for people with amyotrophic lateral sclerosis and their caregivers. *The Journal of Alternative & Complementary Medicine* 20(4), 272–275. doi:10.1089/acm.2013.0268 24328393 PMC3994974

[ref42] Quinn B and Connolly M (2023) Spirituality in palliative care. *BMC Palliative Care* 22(1), 1. doi:10.1186/s12904-022-01116-x 36597069 PMC9811699

[ref43] Rodrigues RMC (2008) Validação da versão em português europeu de questionário de avaliação funcional multidimensional de idosos. *Revista Panamericana de Salud Pública* 23(2), 109–115. doi:10.1590/s1020-49892008000200006 18371281

[ref44] Rosengren K, Gustafsson I and Jarnevi E (2014) Every second counts: Women’s experience of living with ALS in the end-of-life situations. *Home Health Care Management & Practice* 27(2), 76–82. doi:10.1177/1084822314547961

[ref45] Sommers-Spijkerman M, Kavanaugh MS, Kruitwagen-Van Reenen E, et al. (2023) Stigma experienced by ALS/PMA patients and their caregivers: A mixed-methods study. *Amyotrophic Lateral Sclerosis and Frontotemporal Degeneration* 24(3-4), 327–338. doi:10.1080/21678421.2022.2161911 36593637

[ref46] Yuan MM, Peng X, Zeng TY, et al. (2021) The illness experience for people with amyotrophic lateral sclerosis: A qualitative study. *Journal of Clinical Nursing* 30(9-10), 1455–1463. doi:10.1111/jocn.15697 33559184 PMC8248064

